# Functional Amyloids in Pseudomonas aeruginosa Are Essential for the Proteome Modulation That Leads to Pathoadaptation in Pulmonary Niches

**DOI:** 10.1128/spectrum.03071-22

**Published:** 2022-12-08

**Authors:** Ayesha Z. Beg, Faraz Rashid, Absar Talat, Mohd Azam Haseen, Nadeem Raza, Kafil Akhtar, Morten Kam Dahl Dueholm, Asad U. Khan

**Affiliations:** a Medical Microbiology Lab, Interdisciplinary Biotechnology Unit, Aligarh Muslim University, Aligarh, Uttar Pradesh, India; b Henry Ford Health System Detroit, Michigan, USA; c Department of Cardiothoracic Surgery, Jawaharlal Nehru Medical College, Aligarh Muslim University, Aligarh, Uttar Pradesh, India; d Department of Anaesthesiology, Jawaharlal Nehru Medical College, Aligarh Muslim University, Aligarh, Uttar Pradesh, India; e Pathology Department, Jawaharlal Nehru Medical College, Aligarh Muslim University, Aligarh, Uttar Pradesh, India; f Center for Microbial Communities, Department of Chemistry and Bioscience, Aalborg University, Aalborg, Denmark; University of Guelph

**Keywords:** Fap, *Pseudomonas aeruginosa*, amyloid, antimicrobial resistance, biofilms

## Abstract

Persistence and survival of Pseudomonas aeruginosa in chronic lung infections is closely linked to the biofilm lifestyle. One biofilm component, functional amyloid of P. aeruginosa (Fap), imparts structural adaptations for biofilms; however, the role of Fap in pathogenesis is still unclear. Conservation of the *fap* operon encoding Fap and P. aeruginosa being an opportunistic pathogen of lung infections prompted us to explore its role in lung infection. We found that Fap is essential for establishment of lung infection in rats, as its genetic exclusion led to mild focal infection with quick resolution. Moreover, without an underlying cystic fibrosis (CF) genetic disorder, overexpression of Fap reproduced the CF pathotype. The molecular basis of Fap-mediated pulmonary adaptation was explored through surface-associated proteomics *in vitro*. Differential proteomics positively associated Fap expression with activation of known proteins related to pulmonary pathoadaptation, attachment, and biofilm fitness. The aggregative bacterial phenotype in the pulmonary niche correlated with Fap-influenced activation of biofilm sustainability regulators and stress response regulators that favored persistence-mediated establishment of pulmonary infection. Fap overexpression upregulated proteins that are abundant in the proteome of P. aeruginosa in colonizing CF lungs. Planktonic lifestyle, defects in anaerobic pathway, and neutrophilic evasion were key factors in the absence of Fap that impaired establishment of infection. We concluded that Fap is essential for cellular equilibration to establish pulmonary infection. Amyloid-induced bacterial aggregation subverted the immune response, leading to chronic infection by collaterally damaging tissue and reinforcing bacterial persistence.

**IMPORTANCE** Pseudomonas aeruginosa is inextricably linked with chronic lung infections. In this study, the well-conserved Fap operon was found to be essential for pathoadaptation in pulmonary infection in a rat lung model. Moreover, the presence of Fap increased pathogenesis and biofilm sustainability by modulating bacterial physiology. Hence, a pathoadaptive role of Fap in pulmonary infections can be exploited for clinical application by targeting amyloids. Furthermore, genetic conservation and extracellular exposure of Fap make it a commendable target for such interventions.

## INTRODUCTION

Pseudomonas aeruginosa is a multidrug-resistant bacterium associated with morbidity and mortality of patients with opportunistic nosocomial infections (1–3). P. aeruginosa frequently causes chronic lung infections, chronic wound infections, and medical device-facilitated infections (2, 4, 5). P. aeruginosa is inseparably linked with cystic fibrosis (CF), a life-shortening genetic disorder characterized by mucus-mediated pulmonary obstruction and changes in lung architecture (6). Reportedly, almost three out of four adults with CF and approximately 98% of young CF patients are predominantly infected by P. aeruginosa (7). In the long run, aggressive antibiotic regimens fail to eradicate Pseudomonas colonization of CF lungs due to its persistent lifestyle in chronic infections (8). The premises of P. aeruginosa survival in CF lungs depends on host-pathogen interactions, where biofilm lifestyle obscures bacterial clearance by immune cells (9). Furthermore, biofilms impede the neutrophil-mediated response and generate a hyperinflammatory cycle, which leads to development of chronic infection (10).

Recent evidence has shown the biofilm lifestyle or aggregative phenotype is predominantly found in chronic pulmonary infections (11). The extracellular polymeric substance matrix impairs the response of the immune system and effectiveness of antibiotic regimens. The aforementioned scenario has directed a focus toward exploring targets involved in biofilm-mediated pathoadaptation (12). A biofilm is an adherent meshwork comprising exopolysaccharides, extracellular DNA, proteins (e.g., lectins, adhesins, and amyloids), and lipids (13–15). One of the biofilm components produced by P. aeruginosa is the Fap amyloids, which provide hydrophobicity and mechanical robustness to the biofilm (16). Fap also serves as a reservoir for retaining quorum-sensing molecules (17). Moreover, bioinformatic analysis of sequenced strain genomes has revealed that the Fap system is conserved in many genera and is present in almost all P. aeruginosa strains (18–20). Recent evidence associated other bacterial amyloids with pathologies associated with cognitive function, inflammatory responses, and pulmonary functions (21–24). However, similar effects have not been explored for Fap amyloids.

The known effects of Fap on biofilm properties, in combination with the conservation and sophisticated assembly of the *fap* genes into an operon, prompted us to explore its essentiality in pathoadaptation by P. aeruginosa (16, 17, 19). The P. aeruginosa PAO1 wild-type strain (PAO1 wt), a Fap deletion strain (PAO1 Δ*fap*), and a Fap overexpression strain (PAO1 pFap) derivative were studied in a rat model of lung infection to infer the role of Fap-mediated adaptation in establishment of lung infection. An *in vivo* lung infection model was used to describe the effect of Fap on infection pathophysiology and host immune response. The molecular basis of Fap-mediated adaptations associated with attachment and biofilm sustainability were explored by *in vitro* surface-associated proteomics using a glass wool model (25, 26). The glass wool model was used to mimic the lung environment, as it provides a low-shear environment, with interstrand cellular movement and interchange of nutrients and oxygen (25, 26). Although the current *in vitro* biofilm models are limited by the absence of host factors, they are suitable for helping in understanding bacterial physiology. Surface-associated proteomics provided insight into Fap expression-influenced regulation of previously known pathoadaptive and cellular fitness proteins that account for generation of histopathology.

## RESULTS

### Effects of PAO1 Fap variants on biofilm formation and human whole blood.

**FIG 1:**
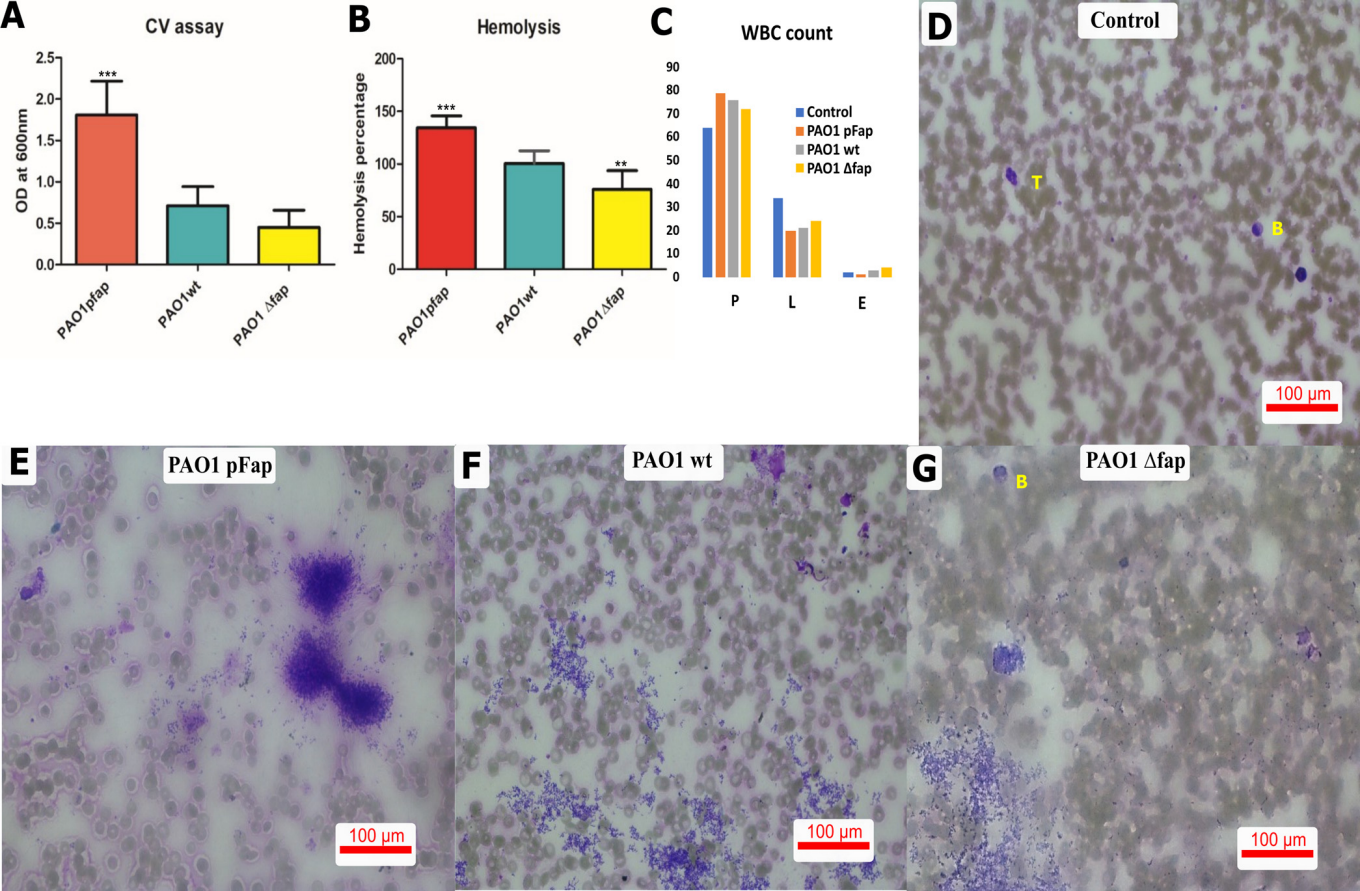
*In vitro* biofilm formation and pathogen-blood studies. (A) Relative quantification of biofilm formation in the CV assay indicated that pFap showed significant biofilm formation with respect to wt, and the group with Fap deleted showed decreased biofilm formation. (B) Hemolysis profiles of different Fap groups. (C) White blood cell (WBC) counts of different groups. P, polymorphonuclear leukocytes (PMN); L, lymphocytes; E, eosinophils. Significant hemolysis was observed in the pfap and Δ*fap* groups compared to the wild type. Other images show the peripheral blood smear of HWB infected with P. aeruginosa *fap* variants. (D) The control image shows bilobed (B) and trilobed (T) PMNs and red blood cells. (E) For PAO1 pFap, PMNs showed moderate (2+) to intense (3+) bacteremia; (F) PAO1 wt, the image shows PMN leukocytosis and moderate (2+) bacteremia; (G) PAO1 *Δfap*, bilobed leukocytes (B) were evident with mild to moderate bacteremia. The assay results were subjected to a one-way ANOVA and are presented as means ± SD.

The relative biofilm quantification by a crystal violet (CV) assay showed highest biofilm formation by PAO1 pFap, followed by PAO1 wt and finally PAO1 Δ*fap* (Fig. 1A). The hemolytic assay results were positively correlated with biofilm formation ability, demonstrating highest hemolysis by PAO1 pFap, followed by PAO1 wt and then PAO1 Δ*fap* (Fig. 1B). The human whole-blood (HWB) assay demonstrated a slight difference in the numbers of polymorphonuclear leukocytes (PMNs), lymphocytes, and eosinophils (Fig. 1C), but a distinct infection profile was observed (Fig. 1D to G). The HWB infected by bacteria showed persistence of aggregated colonies for PAO1 pFap (marked bacteremia [score of 3+]) (Fig. 1E), PAO1 wt (moderate bacteremia [2+]) (Fig. 1F), and PAO1 Δ*fap* (mild bacteremia [+]) (Fig. 1G).

### Effects of Fap system in *in vivo* rat lung bacterial colonization.

**FIG 2:**
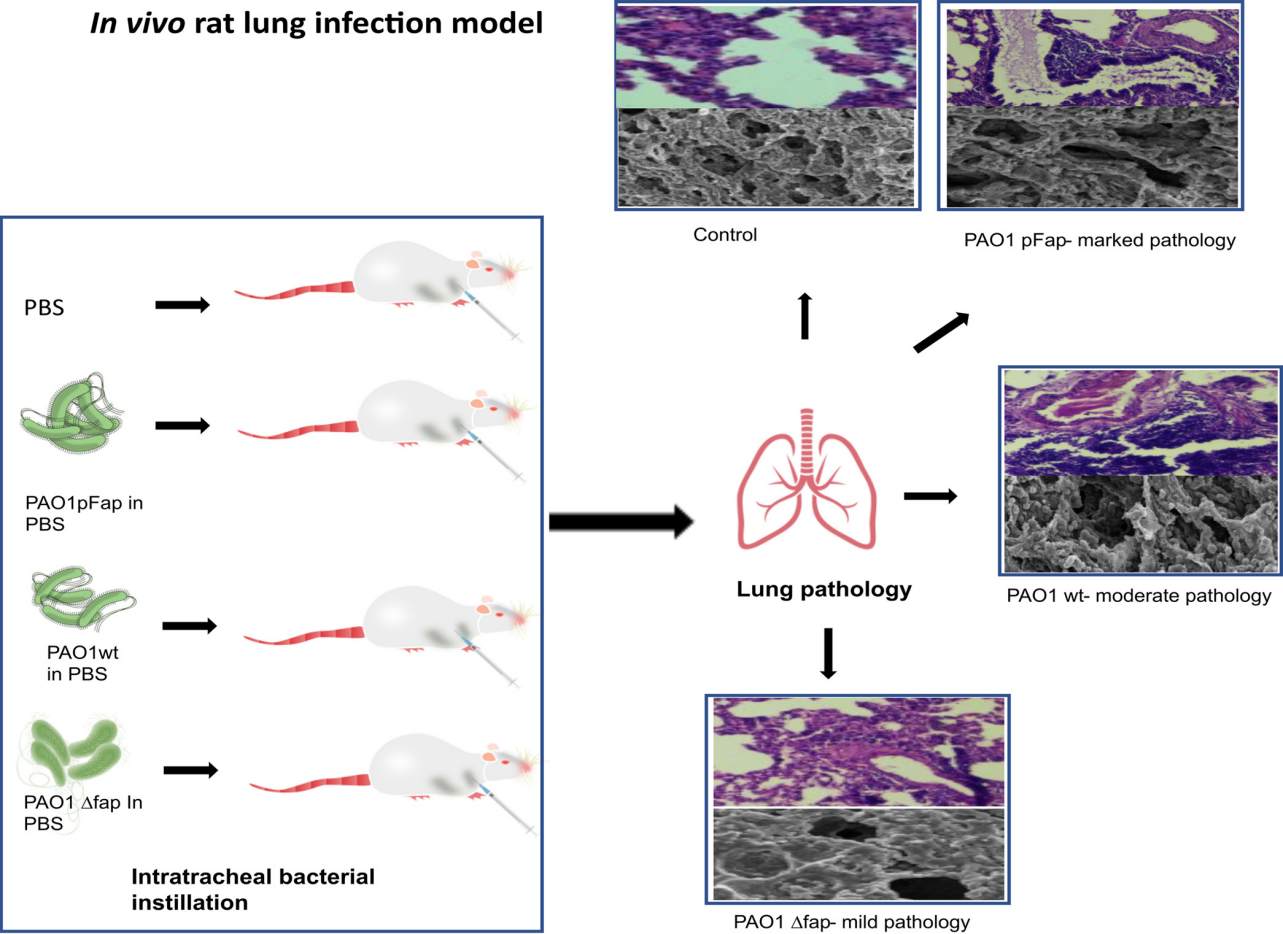
Schematic workflow for the rat lung infection model. (Left) Intratracheal bacterial instillation of different Fap variants in Wistar rats. (Right) Histopathological studies to determine infection progression.

The implication of Fap in generation of lung infection was explored using *in vivo* rat models (Fig. 2). The PAO1 wt, PAO1 *Δfap*, and PAO1 pFap variants were inoculated into the lungs of rats through tracheostomy, and bacterial colonization and the progression of infection through histopathology were observed on days 3, 7, and 11.

**FIG 3:**
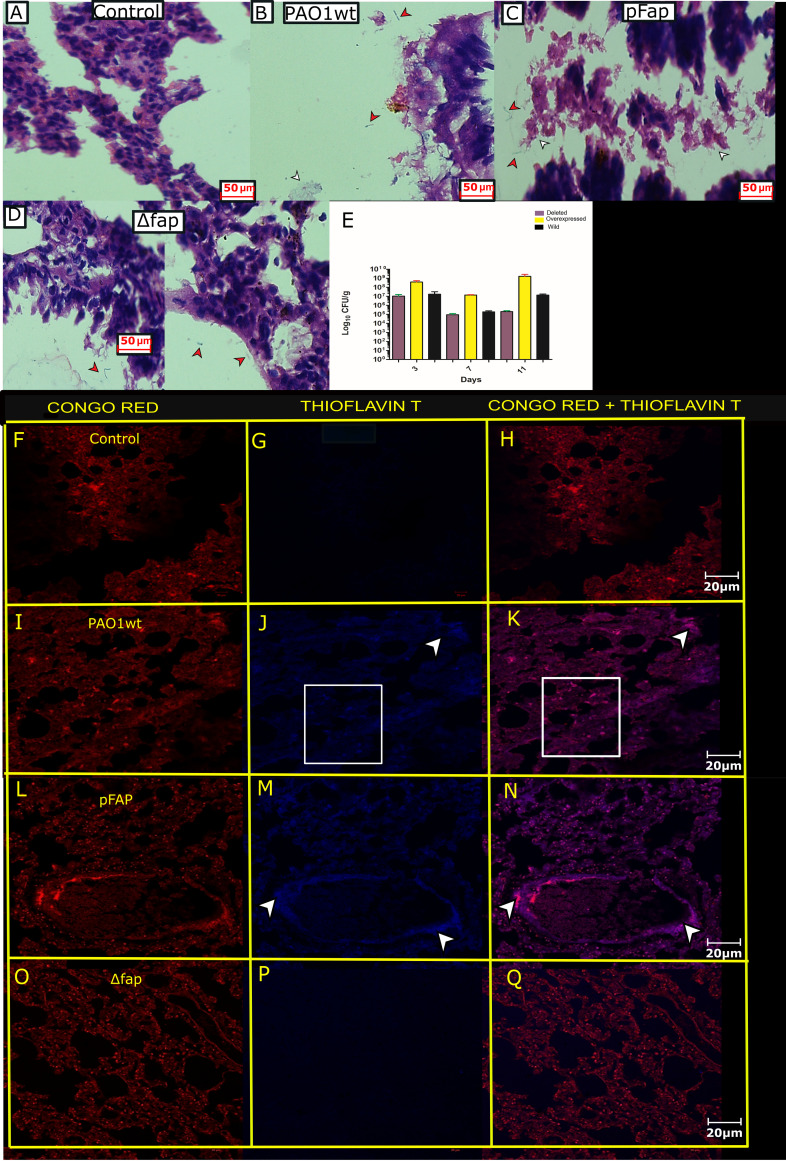
Bacterial colonization and detection of amyloid produced by bacteria in tissue sections. (A) Control H&E-stained lung tissue. (B) Lung tissue infected with the wild type as dispersed bacteria (red arrows) and aggregated form (white arrows) were visualized. (C) Lung tissue infected with pfap. Bacteria are seen in the aggregated mesh (white arrows) and free form (red arrows). (D) Lung tissue infected with *fap*-deleted bacteria found localized in alveolar space (red arrows). (E) CFU per gram of lung for the different variants along the timeline of the experiments. (F) Control Congo red-stained lung tissue. (G) ThT-stained tissue. (H) Overlapped Congo red and ThT stain. (I) Lung tissue of the wild type stained with Congo red. (J) Arrow and box representing dense and dispersed amyloid deposits. (K) Overlapped stains (pink) showing amyloid deposits, indicate by the box and arrow. (L) Congo red-stained overexpressed infected tissue. (M) ThT-stained tissue section, where the arrow indicates fluorescence by amyloid deposits. (N) Overlap of both stains (pink) showing presence of amyloid deposits. (O) Deleted Fap variant, Congo red stained. (P) ThT stained. (Q) Overlapped stains showed absence of amyloid. Magnification: ×40 (A to D) or ×20 (F to Q). Note: CR staining produced background fluorescence of the tissue; therefore, amyloid confirmation was done in combination with ThT.

Hematoxylin and eosin (H&E)-stained lung tissue sections were analyzed to visualize bacterial colonization. The rat groups exposed to PAO1 wt displayed dispersed bacteria and few bacterial aggregates (Fig. 3B), while exposure to PAO1 pFap led to aggregated bacterial colonization resembling a meshwork (Fig. 3C) compared to the control (Fig. 3A). However, fewer localized bacteria in alveolar space were observed in the PAO1 Δ*fap* group (Fig. 3D). After 11 days, bacterial load in the lung tissue indicated a chronic infection for PAO1 pFap, with 10^9^ CFU/g of tissue. The bacterial load for PAO1 wt was10^7^ CFU/g of tissue, while 10^5^ CFU/g of tissue PAO1 Δ*fap* bacterial load was determined (Fig. 3E). To confirm that the improved colonization was related to the expression of amyloid, lung tissues were stained with the amyloid-specific dyes Congo red (CR) and Thioflavin T (ThT). The combination of CR and ThT dyes was selected for amyloid confirmation, as CR staining produced background fluorescence of the tissues. The lung tissue sections colonized with PAO1 wt displayed dispersed amyloid deposits (Fig. 3I to K), while PAO1 pFap showed presence of dense amyloid deposits (Fig. 3L to N). Amyloids were not detected in the infection negative control (Fig. 3F to H) or in lungs infected with PAO1 Δ*fap* (Fig. 3O to Q).

### Effects of Fap on infection pathology of lungs and cytokine response.

**FIG 4:**
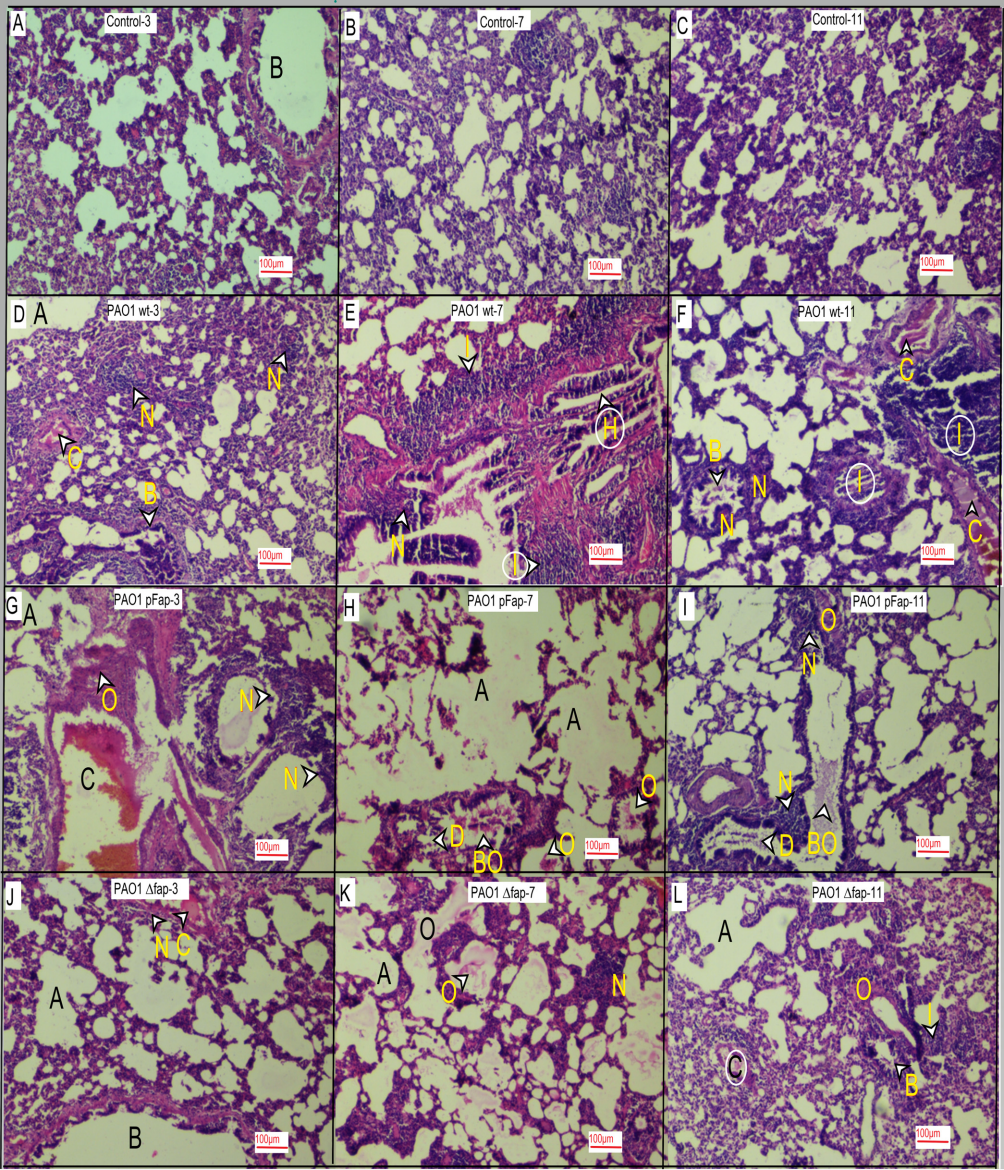
Histopathological analysis of lung tissues. (A) Control, day 3. Tissue section shows normal alveolar spaces and a bronchiole with normal stratified columnar epithelium with thin muscle layer. (B) Control, day 7. Tissue sections show normal alveolar spaces with normal capillaries in the alveolar septum. (C) Control, day 11. Tissue sections show normal alveolar spaces with normal capillaries in the alveolar septum. (D) Wild type, day 3. Section shows mildly (+) dilated alveoli (A), mild vascular congestion (C), dense moderate (2+) neutrophilic aggregates (N), and moderate (2+) bronchial neutrophilic infiltrate (B). (E) Wild type, day 7. Tissue sections show mild (+) dilated alveoli (A), marked (3+) dense peribronchial neutrophilic infiltrate (N), marked (3+) alveolar inflammation (I), and bronchial epithelial hyperplasia. (F) Wild type, day 11. Section shows mildly (+) dilated alveoli (A), moderate vascular congestion (C), marked (3+) interstitial inflammation (I), bronchial epithelium destruction (B), and mild peribronchial neutrophilic infiltrate (N). (G) Overexpressed *fap*, day 3. Section shows moderately (2+) dilated alveoli (A), marked (3+) vascular congestion (C), marked (3+) peribronchial neutrophilic infiltrate (N), interstitial inflammation (I), and alveolar edema (O). (H) Overexpressed *fap*, day 7. Section shows marked (3+) alveolar destruction and dilation (A) turning into alveolar sacs with alveolar edema (O), bronchial destruction (D), and bronchial edema (BO). (I) Overexpressed *fap*, day 11. Section shows moderately (2+) to markedly (3+) dilated alveoli (A), alveolar edema (O), bronchial destruction (D), edema (BO), and focal marked (3+) neutrophilic infiltrate around bronchi (N). (J) Deleted *fap*, day 3. Tissue section shows mildly (+) dilated alveoli, focal neutrophilic infiltrate (N), mild (+) vascular congestion (C), and mildly (+) dilated bronchi (B). (K) Deleted *fap*, day 7. Section shows mildly (+) to moderately (2+) dilated alveoli (A), alveolar edema (O), mild peribronchial acute inflammation (B) (I), and mild (+) focal interstitial neutrophilic infiltrate (N). (L) Deleted *fap*, day 11. Section shows mildly dilated (+) alveoli (A), mild (+) vascular congestion (C), interstitial edema (O), mild (+) interstitial neutrophilic infiltrate (I), and mild (+) peribronchial inflammation (B). Magnification (all images), ×10.

**(i) Histopathological analysis.** The H&E-stained lung tissue sections were analyzed, and pathology was graded as described in Materials and Methods. The control group lung sections from days 3, 7, and 11 demonstrated normal lung tissues (Fig. 4A to C). At day 3, the PAO1 wt-infected group showed mild alveolar dilation, vascular congestion, dense moderate neutrophilic aggregates, and peribronchial neutrophilic infiltrates (Fig. 4D). On day 7, peribronchial neutrophil infiltration increased along with bronchial hyperplasia and marked alveolar inflammation (Fig. 4E). At day 11, moderate congestion with marked interstitial inflammation and bronchial epithelium destruction were observed (Fig. 4F). The infection progression pattern of the PAO1 pFap-infected group on day 3 displayed marked neutrophilic infiltration accompanied by inflammation, vascular congestion, and moderate alveolar dilation (Fig. 4G). On day 7, apart from persistent neutrophilic inflammation, characteristic emphysema with alveolar and bronchial destruction (27) and CF-like secretion in bronchial spaces were observed (Fig. 4H). On day 11, apart from chronic neutrophilic inflammation, persistent emphysema, increased alveolar dilation and edema, bronchiectasis with bronchial dilation, and CF-like secretion were also apparent (6) (Fig. 4I). The infection progression of the PAO1*Δfap*-infected group on day 3 showed mildly dilated bronchi and alveoli with focal neutrophilic infiltrates (Fig. 4J). Focal neutrophilic infiltration with mild acute peribronchial inflammation was observed on day 7 (Fig. 4K). Mild focal pathologies, like vascular congestion, neutrophilic infiltrates, and peribronchial inflammation, persisted until day 11 (Fig. 4L).

**FIG 5:**
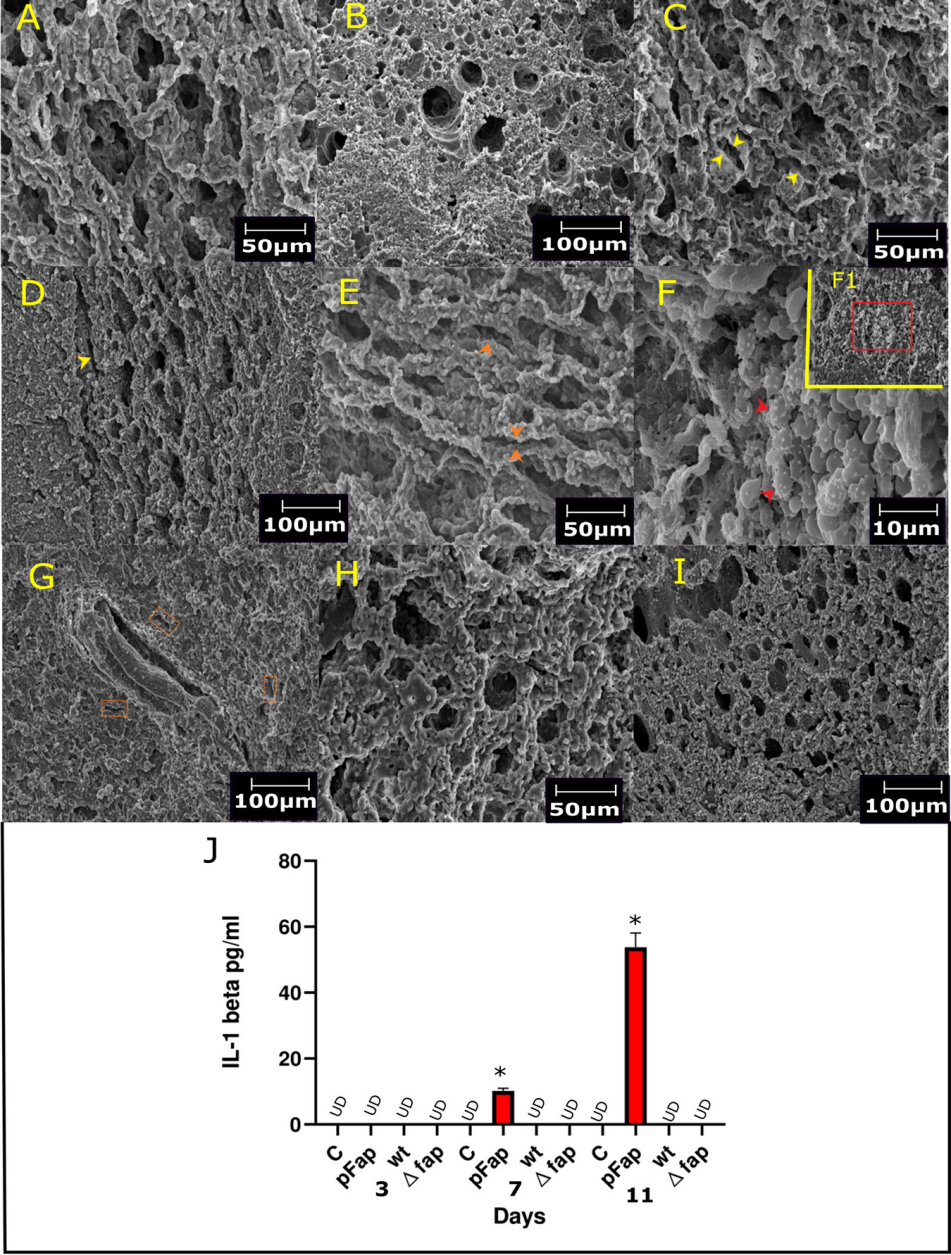
Ultrastructure of lung tissue and systemic levels of cytokines. (A) Day 3, control: normal alveolar space was observed. (B) Day 11, control: normal lung architecture. (C) Day 3, PAO1 wt: changes in airway architecture (yellow arrows). (D) Day 11, PAO1 wt: moderate narrowing of alveolar lumen (yellow arrow). (E) Day 3, PAO1 pFap: extensive alveolar space collapse (orange arrows). (F) Day 7, PAO1 pFap: influx of acanthocytes (red arrow), F1 broader view demonstrating marked presence of acanthocytes (red box). (G) Day 11, PAO1 pFap: marked collapse and obstruction of airways (orange boxes). (H) Day 3, Fap deleted: no prominent observation was apparent. (I) Day 11, Fap deleted: in comparison with control, no significant observation was made. (J) Cytokine panel, for IL-1β measured for different groups at a certain time point, significant change was observed in the Fap overexpressed group on day 11, while it remained undetectable (UD) in other groups. Magnification: ×500 (A to D, day 3 lung sections); ×2,500 (E, day 7 pFap); ×200 (F to I, day 11 lung sections). Cytokine assays were performed in technical duplicates. Significance and nonsignificance (ns) were determined with respect to PAO1 wt. *, *P* < 0.01.

**(ii) Ultrastructure of infected lungs.** The ultrastructure of lung tissues was assessed by scanning electron microscopy (SEM), where the lung structure of the control group remained normal during the course of experiment (Fig. 5A and B). At day 3, slight narrowing of the alveolar lumen was observed in the PAO1 wt-infected group, which was complemented by fewer acanthocytes on day 7 (Fig. 5C). Narrowed air spaces and poikilocytes persisted until day 11 (Fig. 5D). On day 3, unusual narrowing of the alveolar lumen was observed in the PAO1 pFap-infected group, with an additional surge of acanthocytes on day 7 (Fig. 5E and F) (28). The collapsed alveolar spaces and poikilocytes persisted on day 11 with coalesced alveoli generating large airway lumen spaces (Fig. 5G). There were no distinct changes observable with respect to the control in the PAO1 *Δfap*-infected group during the time course (Fig. 5H and I).

**(iii) Inflammatory response of infection.** Serum cytokine levels of interleukin-6 (IL-6), gamma interferon (IFN-γ), tumor necrosis factor alpha (TNF-α), and IL-1β were assessed in all groups. Increases in IL-1β are correlated with neutrophilic hyperinflammation in chronic infection (10). The IL-1β sera levels in the PAO1 pFap group increased with progression of time compared to other groups (Fig. 5J). The IL-6, IFN-γ, and TNF-α levels in sera were found to be undetectable (29).

### Surface-associated proteomics demonstrated the influence of Fap on bacterial adaptivity and fitness.

**FIG 6:**
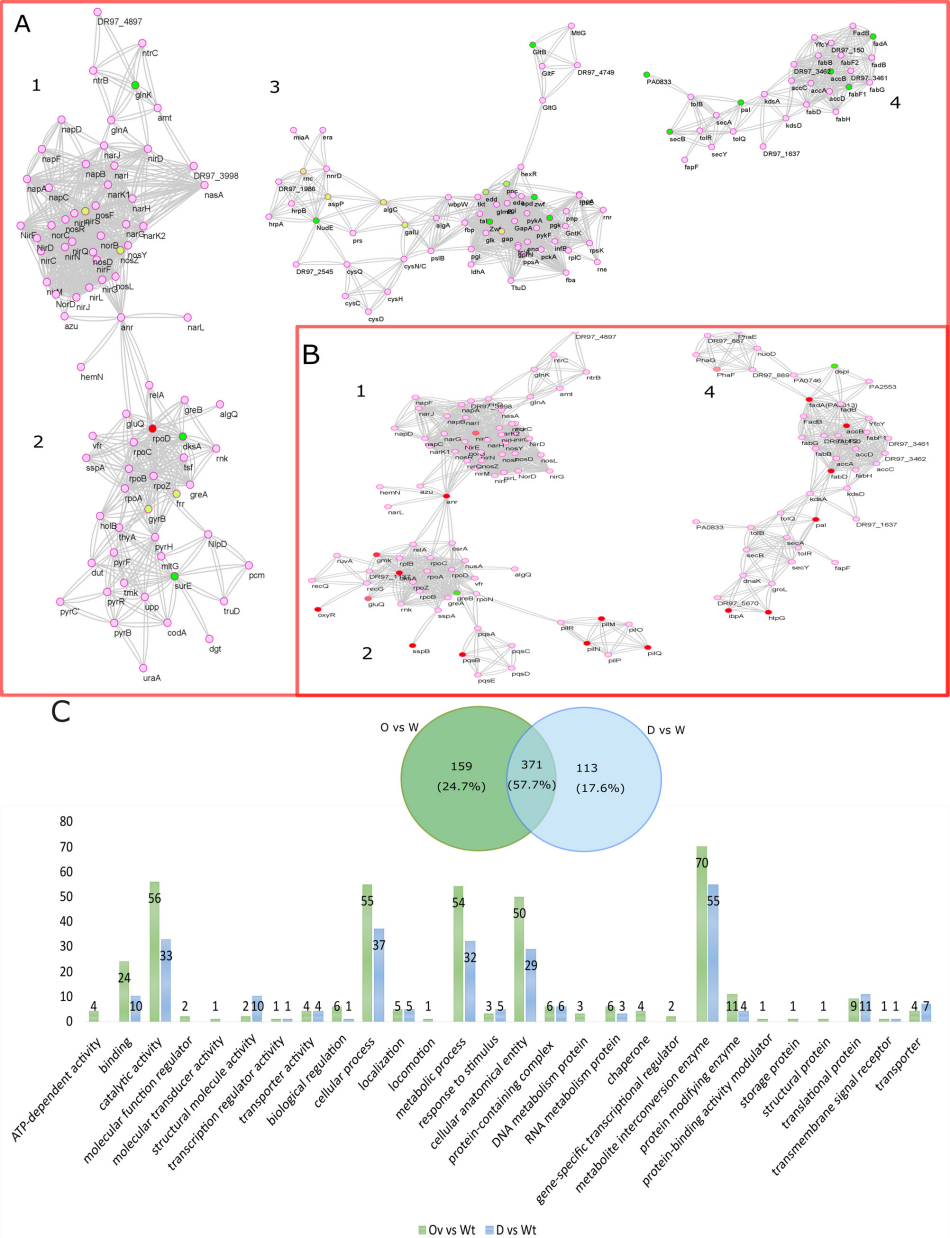
Fap influence protein interaction network and GO classification of differential proteomics. Comparative interactive networks in the presence and absence of Fap system denoting activation (green and yellow nodes based on decreasing values of log-fold change) and suppression (red and salmon nodes based on decreasing values of log-fold change) in critical protein interactomes. (A) Interactome status in overexpressed system. 1, anaerobic pathway and nitrogen regulatory hub; 2, biofilm formation and maintenance hub; 3, glucose and pathoadaptation hub; 4, fatty acid, membrane proteins, and chaperone hub. (B) Interactome status in the absence of Fap. 1, anaerobic pathway and nitrogen regulatory hub; 2, biofilm formation, maintenance, and attachment hub; 4, dispersion, fatty acid, membrane proteins, and chaperone hub. (C) GO classification of proteins exclusive to O versus W and D versus W proteome. Bar graph demonstrates clustering of number of proteins to certain functional class in respective group (O versus W and D versus W is PAO1 pFap versus PAO1 wt and PAO1 *Δfap* versus PAO1 wt).

The effects of the Fap system on the bacterial physiology associated with biofilm sustainability, pathoadaptivity, and cellular fitness were studied through surface-associated proteomics that could provide a link to the pulmonary histopathology. The differential protein expression patterns arising due to bacterial phenotypic heterogeneity were captured by surface-associated proteomics of two groups, the biofilm group and the surface-influenced planktonics (SIP) group (25). The grouping was done as follows: the biofilm group included PAO1 pFap versus PAO1 wt and PAO1 *Δfap* versus PAO1 wt; the SIP group included PAO1 pFap versus PAO1 wt and PAO1 *Δfap* versus PAO1 wt. The different proteomes in the absence and presence of a Fap system showed activation and suppression of critical interactomes involved in biofilm formation and maintenance, anaerobiosis, metabolic competency, cellular integrity, and fitness in protein machinery. Distinct expression of critical interactive domains in PAO1 pFap versus PAO1 wt and PAO1 *Δfap* versus PAO1 wt groups are illustrated in Fig. 6A and B. The comparisons of the PAO1 pFap versus PAO1 wt and PAO1 *Δfap* versus PAO1 wt nonoverlapping proteomes revealed 159 and 113 proteins, respectively, which were enriched in gene ontology (GO) classes (Fig. 6C). The GO classes associated with DNA metabolism, transcription machinery, and ATP-utilizing proteins were exclusive to PAO1 pFap versus PAO1 wt, indicating dynamism in regulatory circuits (Fig. 6C).

**FIG 7:**
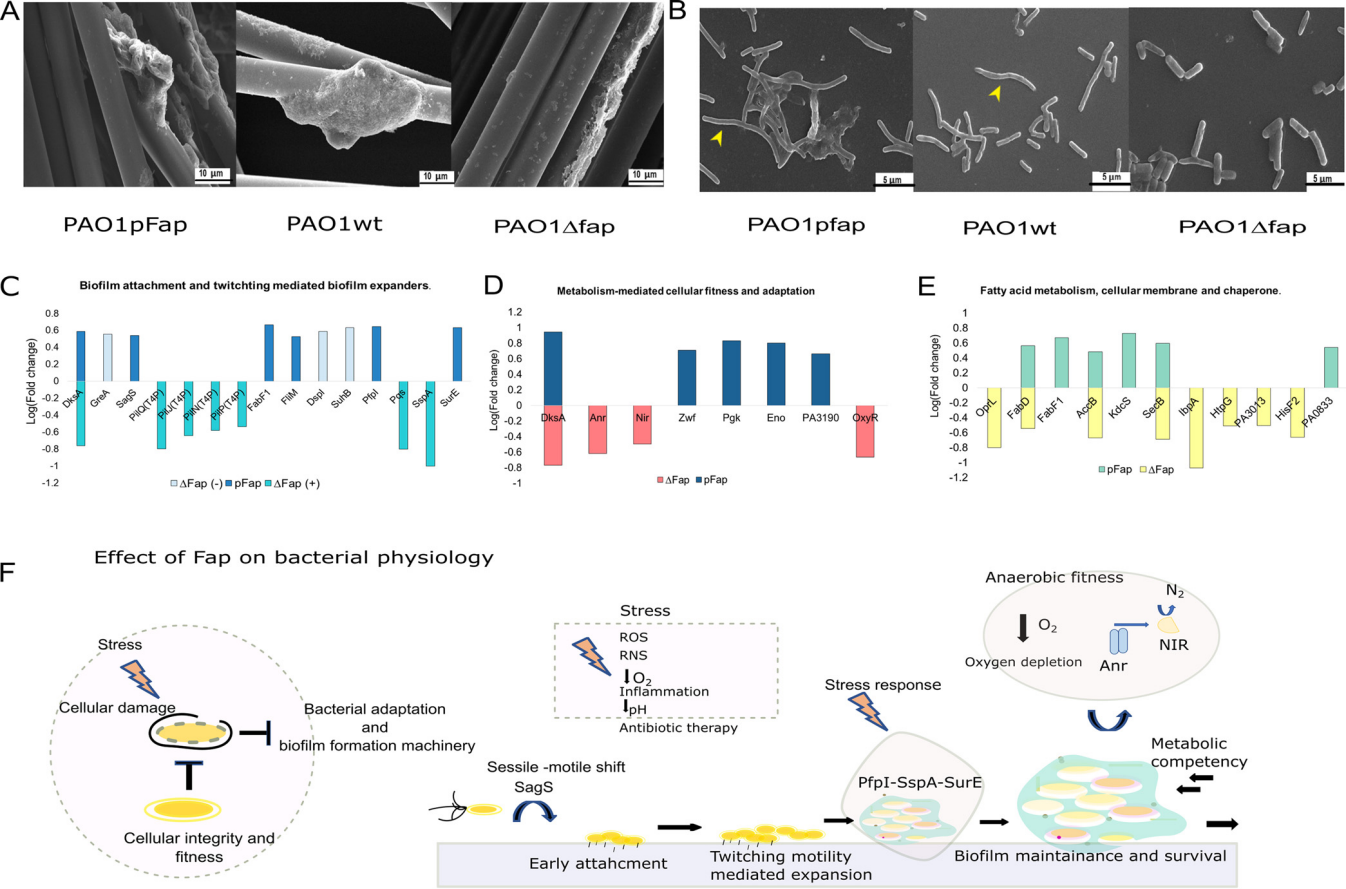
Fap-influenced bacterial phenotypical changes, differentially expressed proteins, and theoretical model demonstrating Fap-mediated adaptations. (A) SEM images of surface-attached biofilm of different Fap variants taken for surface-associated proteomics. (B) SEM images of SIP of different Fap variants taken for proteomics; yellow arrows indicate presence of elongated cellular morphology in PAO1 pFap and PAO1 wt groups, while rod-like morphology was observed in *Δfap*. (C) Proteomics comparison of biofilm attachment and twitching-mediated biofilm expanders, where log (fold change) of important proteins was related to each genetic variant. (D) Metabolism-mediated cellular fitness and adaptation proteins comparison between Fap variants. (E) Important differentially expressed proteins associated with cellular integrity, fatty acid synthesis, and chaperone. (F) Schematic model representing functionality of different protein hubs in biofilm formation and maintenance of biofilm, and also stress response under the influence of Fap expression. *Δfap*(−) represents protein expression that inversely affected biofilm formation, while *Δfap*(+) represents proteins that were positively correlated with biofilm. pfap(+) represents proteins positively affecting biofilm formation.

The SEM images of characteristic biofilm and morphologies of SIP cells of Fap variants subjected to proteomics are shown in Fig. 7A and B and provided correlation with expression of proteins and cellular morphologies. The differential expression of selective proteins that are relevant to pathoadaptation are shown in Fig. 7C to E, and a model elucidating their mechanism in adaptation is presented in Fig. 7F. Furthermore, differentially expressed proteins that demonstrated the linkage between Fap expression with proteome supporting biofilm lifestyles and adaptation to various stresses and the connection of absence of Fap with proteome sustaining planktonic lifestyle with lower cell fitness are discussed in detail below.

### Cellular attachment, biofilm formation, and sustainability.

The Fap-influenced proteins responsible for cellular attachment and biofilm development and maintenance are explained in detail in Fig. 7A and C. Transcription factor DksA (PA4273), a critical part of the bacterial stress response (SR GO category) which regulates quorum sensing (QS GO category), virulence, swarming, and biofilm formation, was upregulated in PAO1 pFap attached cells while downregulated in PAO1 *Δfap* SIP (30). DksA and GreA functionally competed to bind to RNA polymerase and modulate the transcription differently (30). GreA was upregulated in PAO1 *Δfap* SIP, while reciprocal expression was observed in PAO1 pFap SIP.

The T4P responsible for initial attachment and twitching-mediated biofilm expansion was significantly downregulated in PAO1 Δ*fap* SIP (31, 32). The twitching-mediated biofilm expanders PfpI (protease important for survival in reactive oxygen species [ROS]-rich chronic infection niche), FabF1(beta-ketoacyl-acyl carrier protein synthase II), and FliM were upregulated in the PAO1 pFap SIP group (31, 33, 34). The SIP state of PAO1 pFap displayed upregulated sensor-regulator hybrid SagS (PA2824), which promotes surface attachment by activating the motile-sessile switch through small RNA regulation and biofilm sustainability (35). Similarly, PqsB of PqsABCDEF, whose production is central to stationary phase and promotion of biofilm formation, was significantly downregulated in PAO1 Δ*fap* SIP (36).

Likewise, other miscellaneous proteins that affected biofilm sustainability were differentially expressed. The PAO1 *Δfap* SIP displayed significant upregulation of DspI, an enoyl-coenzyme A hydratase or isomerase. DspI is critical for swarming motility and biofilm dispersion but reciprocal to biofilm density (37). Another regulator, SuhB, inversely regulates biofilm formation under the influence of c-di-GMP through the Rsm/Gac pathway (38). In PAO1 *Δfap* SIP, SuhB was upregulated, but in the other group SuhB was downregulated. There was significant downregulation of stringent starvation protein A (SspA) in the PAO1 *Δfap* SIP group; this protein provides adaptation to stringent stress responses in a new environment (39). A nudix pyrophosphatase (PA5176) relieved ROS generation and also contributed toward motility and pathogenicity (40). PA5176 was found to be upregulated ~8-fold in PAO1 pFap attached cells. The PAO1 pFap attached cells displayed upregulation of survival protein E (SurE), which facilitates survival of cells in stationary phase (41).

### Metabolism-mediated cellular fitness and adaptation.

The PAO1 Δ*fap* SIP displayed significant suppression of DksA, Anr, and a nitrite reductase (NIR) protein involved in anaerobic respiration during oxygen stress in lungs (Fig. 7D). Apart from being essential for biofilm in anaerobic state, NIR genes maintained the elongated cell morphology that causes effective cellular clumping leading to robust biofilm formation (Fig. 7B) (42). The suppression in proteins associated with anaerobiosis in PAO1 *Δfap* SIP explained the rod-shaped cellular morphology (Fig. 7B) (42). The PAO1 wt and PAO1 pFap strains displayed elongated cellular morphology (Fig. 7B), which were supported by a functional anaerobic pathway (42).

PAO1 pFap biofilm showed upregulation of glucose-6-phosphate 1-dehydrogenase (Zwf), phosphoglycerate kinase (Pgk), enolase (Eno), and PA3190, which are associated with glycolytic metabolism (Fig. 7D). Zwf provides fitness in the CF environment and sputum and prevents growth inhibition of Pseudomonas (43), while enolase is critical for lung colonization and osmoprotection (44). Pgk provides ROS-induced metabolic fitness (45), while PA3190 is responsible for the glucose uptake operon (46). A stress response regulator, OxyR, was downregulated in Δ*fap* SIP; this regulator is required for establishing infection and evasion of neutrophils (47) (Fig. 7D).

### Fatty acid metabolism, cellular membrane, and chaperones.

The PAO1 *Δfap* SIP displayed downregulation of multiple proteins associated with synthesis and metabolism of fatty acids and cellular wall components like OprL of Tol-Pal and FabD, AccB, PA3013, and HisF2 (Fig. 7E). The OprL of the Tol-Pal system is involved in biogenesis of the outer membrane, assembly of porins, and transfer of lipopolysaccharides (48). AccB under the influence of SigX maintains the cell envelope integrity (49). FabA is involved in fatty acid metabolism, and PA3013 and HisF2 are associated with lipopolysaccharide biosynthesis (50–52). The “CF-selected proteins” Fab, Acc, and OprL, which facilitate integrity-mediated fitness in the CF lung environment, were upregulated in PAO1 pFap (53). Attached PAO1 pFap showed upregulation of FabF1, which is responsible for appendages and membrane fluidity, and KdsC, which is essential for liposaccharide production responsible for cellular integrity (54, 55). A protease, PA3611, was 10-fold upregulated in PAO1 pFap biofilm, which is associated with lung inflammation and bronchial fibrosis (56). Another CF-select OmpA to -C-like protein (PA0833) was upregulated in PAO1 pFap biofilm; this protein facilitates chronic infection (57). Molecular chaperones like SecB, IbpA, and HtpG were downregulated in attached PAO1 ***Δ****fap* cells (Fig. 7E). SecB exports proteins, and IbpA controls antiaging effects of a subpopulation by maintaining protein degradasome and folding homeostasis (58). HtpG is related to biofilm formation and, importantly, adhesion to surfaces (59).

## DISCUSSION

We initiated this study to explore the significance of the conservation and evolution of the Fap system into an operon by P. aeruginosa (18, 19). To address this question, a *in vivo* rat lung infection model and surface-associated proteomics were utilized. The *in vivo* study demonstrated essentiality of P. aeruginosa in generating pulmonary infection. Differential proteomics of P. aeruginosa PAO1 Fap variants indicated selective expression of pathoadaptive proteins that correlated with histopathological manifestations. Moreover, the surface-associated proteomics study delineated that Fap expression necessitates global proteome changes associated with mechano-sensing and physiological modulation, which reinforce P. aeruginosa biofilm sustainability. Reportedly, an exopolysaccharide-driven aggregated phenotype akin to biofilm is found in CF sputum and obstructs innate defenses (60). Marked to moderate bacterial aggregation was revealed by amyloid-producing strains in a pulmonary environment and the *in vitro* whole-blood system. At a molecular level, two critical regulators were responsible for the biofilm formation and continuum, as DksA and SagS were positively associated with Fap expression. DksA via ppGpp regulates the stringent stress response and cellular persistence, while SagS, necessary for chronic infections, controls the phosphorylation cascade that generates continuous biofilm sustainability (30, 35, 61). The bacterial aggregates akin to *in vitro* biofilm downregulate virulence factors, which consequentially decrease host immunostimulation (62). The aggregative phenotype evokes the innate neutrophilic response, but the biofilm matrix shields from phagocytosis and host antimicrobials (62). Moreover, the size of aggregates impedes phagocytosis as demonstrated by *in vitro* HWB infection. Recruited neutrophils produce elevated neutrophil elastase, which further compromises bacterial clearance and causes airway destruction, as evident in histopathological observations (6, 63).

The neutrophilic activities deplete the limited oxygen supply in the pulmonary environment for P. aeruginosa (10, 64). This situation pushes the “obligate aerobe” to activate denitrification pathways and opt for an anaerobic lifestyle (10). The rod-like PAO1 *Δfap* phenotype, with significant downregulation of Anr and DksA, suggests a defect in anaerobic pathoadaptation. Furthermore, an interactive network showed that Anr, Nir, and Fap regulons were interconnected (data not shown). The absence of Fap suppressed the interactomes associated with biofilm formation, stress response, and cellular integrity in PAO1 *Δfap* and effaced matrix-mediated defense, leading to decreased pathogenesis. The DksA involvement in infection establishment and motility regulation explained why its downregulation in PAO1 *Δfap* affected initial attachment to lung surfaces (65). Higher DspI and SuhB expression levels shifted PAO1 *Δfap* to planktonic lifestyle, making it susceptible to host immune response, bacterial clearance, and mild bacteremia in the HWB model. Furthermore, the suppressed chaperone activity was associated with the dysregulated protein homeostasis necessary for cellular fitness. Moreover, OxyR-mediated protection against bacterial clearance and neutrophil evasion was compromised due to its suppression in PAO1 *Δfap* (47).

Interestingly, the PAO1 pFap strain generated an end-stage (late-stage) emphysema CF pathology without underlying genetic disorder. Fap overexpression modulated bacterial physiology akin to CF isolates by upregulating pathoadaptive proteins (CF selected) that can derive nutrition, survive CF airway stress, and persist (53). Upregulation of glucose metabolism proteins Zwf, Eno, Pgi, PA3190, and OprB-glucose uptake suggested utilization of increased glucose in airway surface liquid (ASL), as found in CF-related diabetes (CFRD) (66). Proteins associated with cellular integrity, exopolysaccharide production (AlgC), and osmoprotection (OsmE and BetA) enabled survival and persistence in the mucus-ridden CF pathotype (53). Furthermore, the increased levels of systemic IL-1β in the pFap group suggested activated inflammasomes causing uncontrolled inflammation, compromised bacterial clearance, and development of chronic infection (67).

In conclusion, the Fap system is conserved in P. aeruginosa to provide cellular fitness for lung colonization, establishment of infection, and persistence. Presence of Fap had immunomodulatory consequences that produced hyperinflammation-mediated chronic infection. Moreover, Fap induces expression of “CF-selected” protein, signifying inextricably linkage with CF lung colonizers. Hence, the conservation, extracellular localization, and involvement of Fap in infection make this protein a target for interventions. Further study is warranted to understand host-pathogen interaction and immunological responses activated by the Fap system.

## MATERIALS AND METHODS

### *In vitro* studies.

**(i) Bacterial strains.** P. aeruginosa PAO1 wt (wild-type strain) (68), PAO1 Δ*fap* (Fap operon deleted in PAO1 wt) (69), and PAO1 pFap (Fap operon cloned in a pMMB190Tc vector transformed in PAO1 wt) (69) were grown on Luria-Bertani (LB) agar plates at 37°C. PAO1 pFap was created by transformation of PAO1 wt with the pFap plasmid as previously described (69) and plated on LB plates supplemented with 70 μg/mL tetracycline, 40 μg/mL Congo red, and 20 μg/mL Coomassie brilliant blue G-250. Tetracycline effectively assisted in the selection of the pFap overexpression plasmid in PAO1 pFap colonies, which were further evaluated based on their binding of Congo red and Coomassie brilliant blue (70). Liquid cultures of PAO1 pFap were obtained by inoculating LB medium supplemented with 70 μg/mL tetracycline with a single colony and incubating the culture overnight at 37°C with agitation. Static biofilm relative quantification was determined in a microtiter plate crystal violet (CV) assay for 16 h, and colorimetric quantification was performed at 600 nm (71).

(ii) ***In vitro* whole-blood infection studies.** Human whole blood (HWB) was collected from Blood Bank JNMC, Aligarh following all safety guideline provided by the blood bank. Heparinized blood (90% final blood concentration) was incubated with bacterial inoculum of 10^7^ CFU/mL in Gibco RPMI 1640 medium. The coculture was incubated for 16 h at 37°C augmented with 5% CO_2_ (72). The peripheral blood smear stained with Leishman stain was observed at ×40 magnification by light microscopy. The hemolytic activity of bacteria was carried for 16 h, and hemolysis was determined at 534 nm as previously described (73).

### *In vivo* studies.

**(i) Ethics statement.** The animal experiments were approved by the Institutional Animal Ethical Committee and the Committee for the Purpose of Control and Supervision of Experiments on Animals (reference number 1979/GO/Re/S/17/CPCSEA/20).

**(ii) Animals.** Female Wistar rats purchased from AIIMS (New Delhi, India) were inoculated with Pseudomonas variants as described below. The animals were maintained under controlled temperature (26°C) with 12-h dark-light cycle.

**(iii) Rat lung infection model.** Rats weighing 200 to 250 g (10 to 11 weeks old) were tracheotomized for bacterial instillation under anesthesia (exposure of 5 mL ether in desiccator for 2 min augmented with intramuscular ketamine at 100 mg/kg) (74). A bacterial inoculum size of 200 μL containing 10^7^ CFU of PAO1 wt (group 1), PAO1 pFap (group 2), or PAO1 Δ*fap* (group 3) in phosphate-buffered saline (PBS) was used for instillation. PBS without bacteria was used as a negative control (group 4). The sutured incisions healed without complications. The rats were allowed to recover, and the infection progression was monitored after 3, 7, and 11 days. Three rats from each group were sacrificed at the aforementioned times, and the lungs were removed aseptically. Tissue homogenates of the lungs were serially diluted and 100-μL samples plated on Mueller-Hinton agar plates to determine the bacterial load in CFU per gram of organ. The remaining lung tissues were fixed for histopathological analyses (75, 76).

**(iv) Cytokine assays.** The preinstillation and postinstillation sera were collected and stored at −20°C until analyzed. The following kits were used to assess the cytokines by enzyme-linked immunosorbent assay (ELISA), all obtained from Elabscience: IL-6 (catalog number E-EL-R0015), IFN-γ (catalog number E-EL-R0009), IL-1β (catalog number E-EL-R0012), and RayBio TNF-α (catalog number ELR-TNFa-1).

**(v) Histopathological sample preparation.** Lung tissues were fixed in 10% neutral buffered saline and embedded in paraffin blocks. Tissues sections of 5 μm were prepared as previously described (77). These sections were stained with hematoxylin and eosin and then mounted by using diphenylxylene (DPX). The slides were visualized at ×10 and ×40 magnification. Histopathological scoring of lung tissues was done by grades: marked (3+), moderate (2+), or mild (+), based on degree of pathological manifestation and amount of tissue affected (78).

**(vi) SEM.** The lung tissues were prepared for SEM as previously described (76). The sections were mounted on sample stabs and coated with gold-palladium. Samples were analyzed on three magnifications: ×200, ×500, and ×2,500.

**(vii) CLSM.** For confocal laser scanning microscopy (CLSM), the tissue sections were cut at 8 μm and subsequently deparaffinized by 100% xylene. The tissue sections were stained by Congo red and Thioflavin T as previously described (79, 80). A Fluoview FV1000 CLSM (Olympus, Tokyo, Japan) was used to analyze samples at the excitation wavelengths of 561 nm and 405 nm for Congo red and Thioflavin T, respectively. The emission wavelengths were set at 580 nm and 450 nm for Congo red and Thioflavin T, respectively.

### Surface-associated proteomics study.

**(i) Bacterial strains and growth conditions.** Colonies of PAO1 wt, PAO1 Δ*fap*, and PAO1 pFap grown on LB agar plates were used to inoculate colonization factor antigen (CFA) medium (70) and create overnight cultures. The medium of PAO1 pFap was supplemented with 70 μg/mL tetracycline to maintain the plasmid. A 10% inoculum of each variant was seeded separately into 300 mL CFA containing 3 g glass wool (Merck Millipore catalog number 104086) that covered the base area of a 500-mL Erlenmeyer flask, as previously described (25). All the Fap variant cultures were induced with 0.8 mM isopropyl-β-d-thiogalactopyranoside (IPTG) for PAO1 pFap after 5 h, and all cultures were grown for 16 h, 18 h, and 22 h to obtain different biofilm states.

**(ii) Scanning electron microscopy and confocal microscopy.** The glass wool samples treated with 2% formaldehyde and 2.5% glutaraldehyde solution in PBS were incubated for 1 h at 4°C, followed by dehydration by an increasing ethanol gradient (20 to 100%). The samples were dried, sputter coated with gold-palladium, and then analyzed with SEM as previously described (81). The SEM images for biofilm and SIP groups were take at ×1,000 and ×5,000, respectively. For confocal fluorescence microscopy, the glass wool samples were stained with propidium iodide and Syto9 and analyzed as previously described (82).

**(iii) Sample preparation for proteomics.** The samples were divided in two categories: (i) cells adhered to glass wool fiber (biofilm mass) and (ii) planktonic cells (for SIP). Prior to sample collection, biofilms at different time points were analyzed via SEM and CLSM. Due to viability issues, biofilm samples from 16 h were studied.

SIP biomass was pelleted by centrifugation at 3,381 × *g* for 10 min and washed with PBS prior to storage at −80°C.

**(iv) Biofilm mass extraction.** The glass wool was washed three times with 50 mL of a 0.85% NaCl solution to remove weakly attached cells. After this initial washing, 50 mL of 0.85% NaCl was added along with 30 g of glass beads (mean diameter, 4 mm). The flasks were agitated for 20 min to detach adherent cells, and liquid was collected. An additional 50 mL of 0.85% NaCl was added to the glass wool and agitation was repeated to detach the remaining adhered cells. The combined cell suspension was pelleted at 3,381 × *g* for 10 min, after which the pellet was washed once in PBS and stored at −80°C as previously described (83).

**(v) Protein extraction.** For protein extraction, the pellet was dissolved in lysis buffer (50 mM Tris-HCl [pH 7.2], 300 mM NaCl, 2 mM dithiothreitol, and 1 mM phenylmethylsulfonyl fluoride) followed by ultrasonication (QSonica Q700; 30 W, pulse on for 20 s and pulse off for 5 s for 15 min at 4°C). The lysate was subjected to centrifugation for (4,200 × *g* for 10 min) to remove cellular debris. The supernatant was subjected to centrifugation (13,523 × *g* for 30 min) to obtain the soluble and insoluble fractions. The insoluble pellet was washed with PBS, redissolved, and boiled in lysis buffer containing 2% (vol/vol) SDS for 20 min. The SDS-PAGE loading buffer (2×) was added to the soluble and insoluble fractions. The quantitated protein samples were loaded in equal amounts on a 12% SDS-PAGE gel. The SDS-PAGE was carried out at a constant voltage (100 V, 1 h). The SDS-PAGE was stained with Coomassie R250, and the sample lanes were cut and further excised in 5 pieces. In-gel trypsin digestion was performed as previously described (84), and samples were stored at −20°C until analyzed by mass spectrometer.

### Mass spectrometry.

**(i) Identification.** *(a) Shotgun analysis for protein identification and its acquisition parameters.* An information-dependent acquisition (IDA) process was utilized, in which the MS1 scan determined the mass-to-charge ratio (*m/z*) and the abundance of ions that entered the mass spectrometer, followed by tandem mass spectrometry (MS/MS) fragmentation from a subset of detected peaks (85). The setup was as follows: one full MS scan (*m/z* 350 to 1,250) with accumulation time of 250 ms, followed by 35 data-dependent MS/MS spectra (*m/z* 100 to 1,800) with an accumulation time of 50 ms in each cycle and a total cycle time of ~2.5 to 3.0 s.

Tryptic peptides suspended in solvent A (2% [vol/vol] acetonitrile, 0.1% [vol/vol] formic acid in water) were analyzed with a Sciex 5600+ Triple-time of flight (TOF) mass spectrometer coupled with a BEH column (C_18_, 1.7 μm) in an Exigent ultraperformance liquid chromatography (UPLC) system. The peptides were separated using a 60-min linear acetonitrile gradient starting from 5 to 50% solvent B (98:2% [vol/vol] acetonitrile-water, 0.1% [vol/vol] formic acid) at a flow rate of 300 μL/min with column temperature of 40°C. Data of each fraction were acquired in electrospray ionization positive mode. A maximum of 35 precursor ions with charge states of 2 to 5 that surpassed 100 cps per cycle were selected for fragmentation, and each MS/MS spectrum was accumulated in high-sensitivity mode with dynamic exclusion to 8 to 10 s, as previously described with slight modifications (86). The LC-MS instrument was calibrated prior to each sample set acquisition with Escherichia coli β-galactosidase (BGAL_ECOLI-[P00722]) tryptic digest to maintain the mass within an accuracy of ±2 ppm mass range for MS and MS/MS.

*(b) Shotgun data analysis.* IDA data were processed with Protein Pilot software v. 5.03 (Sciex, Foster City, CA, USA), where a 1% false-discovery rate (FDR) with statistical significance was set for peptides. In brief, the Pseudomonas protein database with 5,586 protein entries (www.pseudomonas.com assembly accession number GCF_000006765.1) was used for protein identification.

**(i) Quantification.** *(a) Data-independent acquisition SWATH and its acquisition parameters.* SWATH is a data-independent acquisition (DIA) method was used for quantification which depends on a previously generated peptide spectral library by shotgun proteomics (87).

First, protein digests (5 to 10 μg) of all samples were analyzed using the SWATH method in the first experiment. Second, we spiked 1 pmol of tryptic peptides derived from E. coli β-galactosidase (β-Gal) into 5 μg of cell lysate digest. All samples were spiked with the equal concentration of β-Gal protein. The β-Gal-containing samples were analyzed by SWATH as well.

Equal quantities of proteins samples digested by trypsin were subjected to DIA (SWATH).The peptides were analyzed using a Sciex Triple TOF 5600 mass spectrometer (SCIEX, USA) equipped with an Exion UPLC connected to an Acquity Premier Peptide BEH C_18_ column (130 Å, 1.7 μm, 2.1 × 150 mm). SWATH was performed for the individual samples to generate high-quality spectral ion libraries by operating the mass spectrometer with specific parameters as previously described, with slight modification (88). Technical triplicates of SWATH were carried out for each sample in order to perform a *t* test.

*(b) SWATH data analysis.* The identified peptides and their corresponding fragment ions were prepared for subsequent spectral library match in the SWATH peak extraction. The peak extraction and spectral alignment were performed using Peakview software (version 2.2; SCIEX, USA). This information was extracted with the SWATH acquisition MicroApp (SWATH 2.0) in Peakview 2.2 software, during library extraction along with other information that was used for peptide and fragment selection within the MicroApp (SWATH 2.0), five transition ions per peptide (b or y ions) and two peptides per protein to be used, and all shared and modified peptides were excluded from the extraction. Further, retention time was realigned according to the manually selected 5 peptides that constantly had high signal intensities and were distributed along the whole-time chromatographic axis. All the quantified peptides were required to have FDRs of <1%. After normalization, principal-component analysis was performed to check the possible correlated variables within the group. The data were further subjected to analysis with MarkerView software (version 1.3.1; SCIEX, USA) for statistical data interpretation. In MarkerView, the results were shown as three output files containing the areas under the curves for the ions, the summed intensity of peptides for protein, and the summed intensity of ions for the peptide. We plotted a volcano curve to determine the statistically significant fold change versus *P* value for each comparative group. Proteins with a significant log fold change of 0.50 (increase or decrease), with a *P* value of ≤0.05 were considered up- or downregulated proteins, respectively.

### Bioinformatic analysis of proteomic data.

The differential abundant proteins were clustered by the following Genetic Ontology classifications: MF, molecular function, i.e., gene products that produce molecular-like activities; BP, biological process, i.e., gene products associated with large biological programs performed by multiple molecular activities; CC, cellular component, i.e., class of gene products associated with anatomical structures of cells; PC, protein classes, i.e., protein families grouping based on their broad functional classes (89). The interaction networks were exported from the STRING 2.0 database to Cytoscape 3.8 (90).

### Statistical analysis.

GraphPad prism 8.0 was used to represent all data in the form of averages ± standard deviations (SD). One-way analysis of variance (ANOVA) was used to compare the multiple means of groups with respect to PAO1 wt. *P* values of <0.05 were considered statistically significant. In biofilm studies, triplicates were considered, while in cytokine assays duplicates were considered.

### Data availability.

The proteomics data are available at the ProteomeXchange Consortium (http://www.proteomexchange.org) through its partner, the PRIDE repository, with project ID PXD033853.
